# A Method for the Pattern Recognition of Acoustic Emission Signals Using Blind Source Separation and a CNN for Online Corrosion Monitoring in Pipelines with Interference from Flow-Induced Noise

**DOI:** 10.3390/s24185991

**Published:** 2024-09-15

**Authors:** Xueqin Wang, Shilin Xu, Ying Zhang, Yun Tu, Mingguo Peng

**Affiliations:** 1 School of Safety Science and Engineering, Changzhou University, Changzhou 213164, China; b22200837006@smail.cczu.edu.cn (X.W.); aezy@cczu.edu.cn (Y.Z.); 2 Key Laboratory of Pressure Systems and Safety, Ministry of Education, East China University of Science and Technology, Shanghai 200237, China; y15230005@mail.ecust.edu.cn (S.X.); ytu@ecust.edu.cn (Y.T.)

**Keywords:** pipeline corrosion, acoustic emission, online monitoring, blind source separation, convolutional neural network

## Abstract

As a critical component in industrial production, pipelines face the risk of failure due to long-term corrosion. In recent years, acoustic emission (AE) technology has demonstrated significant potential in online pipeline monitoring. However, the interference of flow-induced noise seriously hinders the application of acoustic emission technology in pipeline corrosion monitoring. Therefore, a pattern-recognition model for online pipeline AE monitoring signals based on blind source separation (BSS) and a convolutional neural network (CNN) is proposed. First, the singular spectrum analysis (SSA) was employed to transform the original AE signal into multiple observed signals. An independent component analysis (ICA) was then utilized to separate the source signals from the mixed signals. Subsequently, the Hilbert–Huang transform (HHT) was applied to each source signal to obtain a joint time–frequency domain map and to construct and compress it. Finally, the mapping relationship between the pipeline sources and AE signals was established based on the CNN for the precise identification of corrosion signals. The experimental data indicate that when the average amplitude of flow-induced noise signals is within three times that of corrosion signals, the separation of mixed signals is effective, and the overall recognition accuracy of the model exceeds 90%.

## 1. Introduction

Industrial pipelines are essential components of process industries such as the petrochemical and coal chemical industries, maintaining the reliable operation of industrial production and playing a vital role in the national economy and defence construction [1,2]. Due to factors such as the deterioration of crude oil in the chemical industry, extended operating cycles, and harsh and complex operating conditions (high temperatures, high chlorine or sulphur levels, and high acidity levels), some industrial pipelines exhibit increasingly prominent issues such as wall thinning, perforation, or cracking caused by corrosion [3]. This consequently results in the emission of harmful substances, increased maintenance costs, system shutdown losses, and even serious incidents such as combustions and explosions [4], severely impacting the safety of industrial production. Therefore, online monitoring of high-risk industrial pipelines is crucial for preventing further structural failures.

Current commonly used nondestructive testing (NDT) techniques for pipelines include ultrasonic testing [5], 3D laser doppler vibrometers [6], and impulse excitation techniques [7]. However, pipelines that have been in service for a long time, especially those experiencing ongoing corrosion, face challenges in achieving real-time corrosion monitoring. Ultrasonic testing requires a high surface quality of the pipe walls, while 3D laser doppler vibrometry and the impulse excitation technique have limited sensitivity when detecting localized damage such as pitting corrosion. AE technology is a passive NDT method capable of directly measuring elastic waves caused by active defects, offering the potential for monitoring dynamic damage. It is widely applied in structural health monitoring across various fields, such as aerospace [8,9], smart manufacturing [10,11], transportation [12,13], and pressure vessels [14]. Recently, the potential of AE technology in corrosion monitoring has been recognized. Currently recognized sources of pipeline corrosion acoustic emissions include the formation and rupture of hydrogen bubbles and the cracking of passivation films or corrosion products [15,16]. By analysing the acoustic emission signals collected during monitoring, it is possible to determine whether the tested object is undergoing corrosion.

In the field of AE monitoring for pipeline damages, current emphasis is on diagnosing cracking and leakage [17,18,19], with the successful identification and localization of crack or leakage signals in noisy environments being achieved. Current methods for recognizing weak fault signals in noisy environments primarily utilize waveform characteristics or incorporate machine learning techniques. Research has been conducted in the areas of early-stage engine-bearing damages [20], partial discharge faults [21], bridge structural damages [22], and rock fracture monitoring [23] under noisy conditions. To accurately capture AE signals from pipeline corrosion, it is essential to eliminate background noise interference during monitoring and to identify the source of the AE signals. However, methods for detecting pipeline corrosion signals in noisy environments have not yet been fully described due to challenges such as weak energy released from corrosion-related AE sources and flow-induced noise-obscuring corrosion signals [24].

Therefore, a method for the pattern recognition of acoustic emission signals using BSS and a CNN is proposed for online corrosion monitoring in pipelines to mitigate interference from flow-induced noise. Firstly, an online pattern-recognition model for monitoring pipeline corrosion data is presented. It includes a data acquisition module, a signal-separation module, a feature-extraction module, and an acoustic source-recognition module to achieve the identification of corrosion signals in noisy backgrounds. The signal-separation module employs an ICA algorithm based on SSA optimization to separate corrosion and flow-induced noise sources. The feature-extraction module utilizes the HHT to extract time, frequency, and energy three-dimensional spectrograms from the time-domain waveform, compressing the spectrograms to enhance the subsequent recognition efficiency. The acoustic source-recognition module constructs a multilayer CNN with dropout layers to adaptively learn high-dimensional feature representations from input spectrograms for acoustic source recognition. Furthermore, simulated experiments were conducted to verify the effectiveness of the model.

## 2. Theory and Method

### 2.1. The Proposed Model

**Figure 1:**
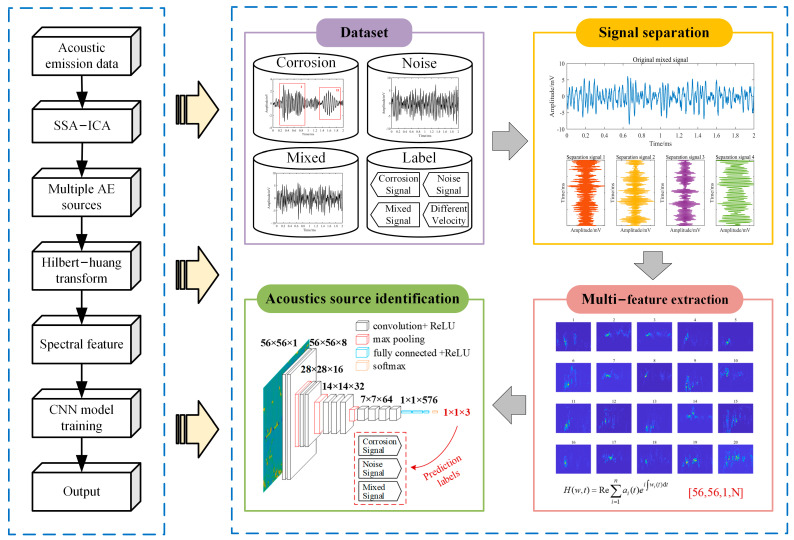
The proposed online model for monitoring pipeline corrosion.

This paper presents a recognition model based on BSS and a CNN, consisting of four modules—data acquisition, signal separation, feature extraction, and acoustic source identification—as illustrated in Figure 1. The model was designed for corrosion monitoring in in-service pipelines under the influence of flow-induced noise. The model collects AE signals during the operation of the pipeline. Through learning from real-time monitoring data, feature extraction, and model training, it can detect the presence of corrosion AE signals amidst continuous flow-induced noise. Compared with other types of noise signals, the interference of flow-induced noise with corrosion signals is more challenging for elimination. This is primarily due to the high-amplitude and wide-frequency spectrum characteristics of flow-induced noise, which renders some simple signal-separation methods ineffective. Moreover, since it is difficult to determine whether a large number of signals during the AE monitoring process are from the same source, decomposition from single-channel signals is required during actual monitoring. In the proposed recognition model, the improved independent component analysis (ICA) method is first applied for blind source separation. The SSA is utilized to decompose the original AE signals into multidimensional observed signals. ICA is then applied to separate these multidimensional observed signals into independent components (ICs), which represent the potential source signals of the mixed signal. Next, the time–frequency information of each IC is compressed into time–frequency maps of size L × L through the Hilbert–Huang transform (HHT), which are combined to reconstruct feature maps. This approach achieves a complete and highly discriminative feature representation of each component of the original signal. Finally, the time–frequency feature maps are input into the CNN model. Through multiple layers of convolutional and pooling layers, the CNN learns the mapping relationship between the AE signals and their internal features. The extracted high-dimensional features are fused through fully connected layers, and the recognition result is output through softmax and classification layers, ultimately determining whether corrosion signals exist in the collected AE data. By identifying the corrosion signal features in the monitoring data, the corrosion state of the pipeline can be timely understood, preventing further damage to the pipeline.

### 2.2. Signal-Separation Module

ICA is a BSS technique widely used for separating source signals from multichannel mixed information [25,26]. ICA decomposes multichannel signals into ICs by utilizing higher-order statistical data. These ICs represent potential source signals that constitute mixed signals. Due to the difficulty in determining whether a large number of signals during the AE monitoring process originate from the same source, decomposition from single-channel signals is required in practical monitoring. Therefore, ICA cannot be directly applied to process AE signals. In this module, we propose an AE signal blind source separation method based on SSA-ICA. The algorithm is implemented in the following steps:

Step 1: Embedding—choose an appropriate window length M to transform the one-dimensional AE signal $X_{N}=\left(x_{1},x_{2},\ldots ,x_{N}\right)$ into a trajectory matrix of size M×K:

(1) $$X=\left[\begin{array}{cccc} x_{1} & x_{2} & \ldots & x_{K}\\ x_{2} & x_{3} & \ldots & x_{K+1}\\ \vdots & \vdots & \ddots & \vdots \\ x_{M} & x_{M+1} & \ldots & x_{N} \end{array}\right]$$

where K=N−M+1.

Step 2: Singular Value Decomposition (SVD)—The covariance matrix is computed by $XX^{T}$, and SVD is performed on the covariance matrix. This yields eigenvalues, $\lambda_{1},\lambda_{2},\ldots ,\lambda_{M}$, and corresponding orthogonal eigenvectors, $U_{1},U_{2},\ldots ,U_{M}$. These eigenvalues and eigenvectors are arranged in ascending order of the eigenvalues. The principal components correspond to the eigenvalues $V_{i}=X^{T}U_{i}\sqrt{\lambda_{i}}$ $\left(i=1,2,\ldots ,d\right)$. The SVD calculation is represented as follows:

(2) $$X=\sum_{i=1}^{d}\sqrt{\lambda_{i}}U_{i}V_{i}^{T}$$

where d is the maximum number of positive eigenvalues.

Step 3: Grouping—For the set obtained from the above decomposition (λ, $U_{i}$, and $V_{i}$), by extracting the frequencies of other components, X is partitioned into mutually disjointed subsets $\left\{I_{1},I_{2},\ldots ,I_{m}\right\}$ to represent different trend changes. Equation (2) can be rewritten in grouped format:

(3) $$X=X_{I_{1}}+X_{I_{2}}+\cdots +X_{I_{m}}$$

where $X_{I_{m}}$ is the feature components related to the original AE signal, and their contribution rates are positively correlated with the singular values.

Step 4: ICA—Search for an appropriate linear transformation for the grouped multidimensional observed signal X, such that the transformed components are statistically independent. The basic model can be represented as follows:

(4) $$X\left(t\right)=AS\left(t\right),t=0,1,2,\cdots$$

where A is the mixing matrix, $S\left(t\right)$ is the matrix of IC vectors, and t is the discrete time sequence.

The IC vector matrix, $S\left(t\right)$, and the mixing matrix, A, are both unknown. In this paper, negative entropy is employed as a measure of non-Gaussianity. The FastICA algorithm, based on fixed-point iterations, is utilized to find the separation matrix, W:

(5) $$H\left(t\right)=WX\left(t\right)=WAS\left(t\right)$$

such that $S\left(t\right)$ and the mixing matrix, $H\left(t\right)$, are as independent as possible. Consequently, the multidimensional ICs of the original AE signals are obtained.

### 2.3. Feature-Extraction Module

To extract the temporal, spectral, and energy information of the acoustic source from the AE waveforms, the HHT is employed in the feature-extraction module. The HHT has been widely used for analysing nonlinear and nonstationary time series, making it highly suitable for extracting time–frequency information from AE signals. The HHT consists of empirical mode decomposition (EMD) and the Hilbert spectral analysis, enabling the acquisition of physically meaningful and instantaneous frequency components in the signal and a subsequent high-resolution time–frequency analysis [27]. The process involves applying EMD to the aforementioned multidimensional ICs, $x\left(t\right)$, adaptively breaking them down into a series of intrinsic mode functions (IMFs) and one residual component, each with different time scales. The decomposition process is represented as follows:

(6) $$x\left(t\right)=\sum_{i=1}^{n}IMF_{i}+r_{n}\left(t\right)$$

where $IMF_{i}$ is the series of intrinsic mode functions obtained through EMD and $r_{n}\left(t\right)$ is the residual component.

Performing the Hilbert transform on the $IMF_{i}$ allows us to obtain a complex IMF signal without causing information loss, expressed as follows:

(7) $$Z_{i}\left(t\right)=a_{i}\left(t\right)e^{j\theta_{i}\left(t\right)}$$

where $a_{i}\left(t\right)$ represents the instantaneous amplitude of the complex IMF, and $\theta_{i}\left(t\right)$ is the instantaneous phase. According to the definition of the instantaneous frequency, the following equation can be obtained:

(8) $$w_{i}\left(t\right)=\frac{d\theta_{i}\left(t\right)}{dt}$$

Therefore, the Hilbert time–frequency spectra of each IC can be obtained, which serve as the input features for the subsequent CNN:

(9) Hw,t=Re∑i=1naitei∫witdt

### 2.4. Acoustic Source-Recognition Module

The acoustic source-recognition module primarily employs a CNN. A CNN is a type of feedforward neural network in which artificial neurons respond to a part of the surrounding units within a certain coverage area, exhibiting excellent performance in processing large-scale image data. The CNN plays a crucial role in the entire identification model, forming a network system with an input layer, convolutional layers, pooling layers, fully connected layers, and an output layer. The input layer is responsible for inputting feature data. In this paper, the input is the feature map obtained after BSS and HHT. Convolutional layers are mainly used for feature extraction. The front-end convolutional layers extract low-level features, while the back-end convolutional layers focus on extracting high-level features (a recombination of low-level features). The pooling layers involve a downsampling process. The fully connected layers are typically located at the end of the entire CNN. They are responsible for transforming the two-dimensional feature map output from the convolutional layers into a one-dimensional vector and finally realize the classification of the pipeline acoustic emission signals. The architecture of the CNN network constructed in this paper is presented in Table 1. The convolutional layers use the nonlinear activation function ReLU. A dropout layer with a dropout rate of 0.5 was added between the second and third convolutional layers to prevent overfitting during model training. The initial learning rate was set to 0.005, and the learning rate was reduced by half every 10 epochs. The number of epochs and batch sizes were set to 50 and 450, respectively. The network training was conducted on an AMD Ryzen 7 5800X3D CPU (Advanced Micro Devices, Inc., Santa Clara, CA, USA) with 32 GB of RAM and an Nvidia GeForce RTX 2080 Super GPU (NVIDIA, Santa Clara, CA, USA).

## 3. AE Monitoring Experimental Setup

### 3.1. Experimental Platform

**Figure 2:**
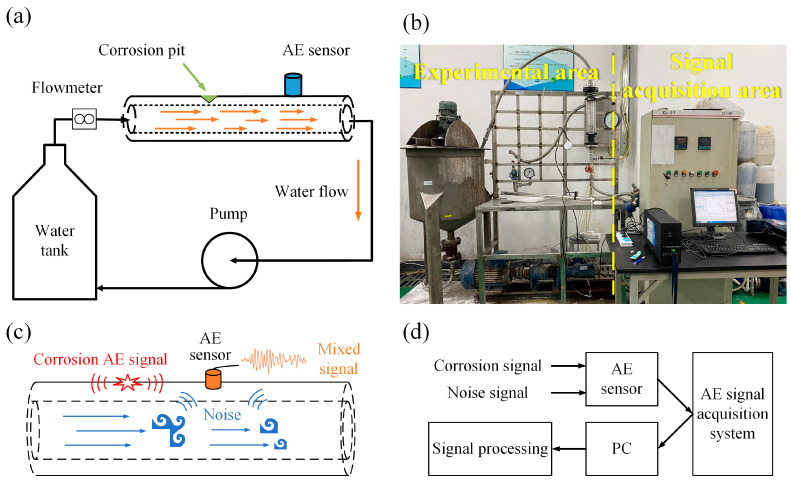
Schematic diagram of the pipeline test bench structure. (**a**) Schematic diagram of the experimental simplification; (**b**) Pipeline corrosion monitoring experimental platform; (**c**) Schematic diagram of the flow-induced noise interference; (**d**) Workflow diagram for data acquisition and processing.

A simulation platform for the detection of liquid pipeline corrosion based on AE signals was established to verify the model. A schematic diagram of the pipeline detection platform comprising the pipeline corrosion experiment area and the AE signal acquisition area is shown in Figure 2. The R3I-AST AE sensor produced by the Physical Acoustics Corporation of America was used for the AE experiments. This sensor features high sensitivity and an integrated front-end amplifier. It is a narrow-band resonant sensor with a maximum peak sensitivity of 120 dB and a resonant frequency range of 10 to 40 kHz. The 40 dB front-end amplifier and filter are fully enclosed within a stainless-steel housing, minimizing RFI/EMI interference. Due to its high sensitivity and low resonance frequency, this sensor is suitable for various applications, including pipelines or tanks in petroleum transport and storage, refining plants, chemical factories, and offshore platforms involving metal and FRP structures.

A sample seamless steel pipe with a diameter of 32 mm, a wall thickness of 3 mm, and a length of 1.5 m was chosen for experimentation, utilizing water as the conveying medium. A water pump provides power to the internal medium of the pipeline, where water enters from one end, flows through, and exits from the other end, creating a circulation that simulates the flow-induced noise generated during pipeline operations. The flow velocity is controlled by a valve, and changes in velocity are monitored by a flow metre. Conical grooves with diameter of 4 mm and depth of 2 mm were prefabricated on the pipe wall for the addition of 6 mol/L of a hydrochloric acid solution, as illustrated in Figure 2a. The AE sensor is installed 10 cm away from the groove and connected to the AE signal acquisition system to output the AE signals, as illustrated in Figure 2b. Figure 2c shows the interference behaviour of the flow-induced noise during the process of pipeline corrosion monitoring. The AE signal from the corroded pipe wall propagates to the AE sensor, and simultaneously, the flow-induced noise generated by the movement of the pipeline medium is also received by the sensor. Therefore, the output of the sensor is a mixed signal from both acoustic sources. The data acquisition and processing workflow is depicted in Figure 2d, utilizing an eight-channel acquisition card from the Physical Acoustics Corporation of America. This card is connected to the PC-side AE acquisition software(Version number: E5.70), with a maximum sampling frequency of up to 10 MHz.

### 3.2. Dataset Construction

To validate the performance of the proposed model, flow-induced noise AE signals, corrosion AE signals, and corrosion AE signals under a flow-induced noise were collected. The experiment was divided into the following three steps:

Step 1: A 6 mol/L hydrochloric acid solution was dripped into the prefabricated groove, and AE signals related to steel pipe corrosion were collected.

Step 2: Without the addition of the hydrochloric acid solution to the prefabricated groove, the water pump was started to allow water to flow inside the pipe. The valve was adjusted to control the flow rate. At different flow rates (0.1 m/s, 0.2 m/s, 0.4 m/s, 0.6 m/s, 0.8 m/s, and 1.0 m/s), flow-induced noise AE signals were collected.

Step 3: The water pump was turned on, a 6 mol/L hydrochloric acid solution was dripped into the prefabricated groove, and mixed AE signals were collected at different flow rates (0.1 m/s, 0.2 m/s, 0.4 m/s, 0.6 m/s, 0.8 m/s, and 1.0 m/s). The hydrochloric acid solution was redispersed each time the flow rate was changed to prevent corrosion signal variations caused by the evaporation of the hydrochloric acid.

During the data collection process, 30 min data were conducted at each flow rate to ensure an adequate number of training samples. A dataset of pipeline corrosion under a flow-induced noise background was constructed, comprising 12 sample categories, as shown in Table 2. The length of each individual signal sampling point was set at 1024, and 2000 signals were extracted for each label. The dataset was then divided into training and validation sets at a 3:1 ratio. The average amplitude of the flow-induced noise signals at different flow rates was greater than that of the corrosion signals and gradually increased with an increasing flow velocity. As the flow velocity increased from 0.1 m/s to 1.0 m/s, the average amplitude of the flow-induced noise signals increased by approximately 10 times. However, the amplitude of the noise signals did not exhibit a linear increase with the flow velocity. In the early stages of increasing the flow velocity, the amplitude of the noise signals increased at a relatively lower rate. When the flow velocity was between 0.1 m/s and 0.6 m/s, the average amplitude of the flow-induced noise was 1 to 4 times that of the corrosion signals. With a further increase in the flow velocity of 0.8 m/s and 1.0 m/s, the average amplitude of the flow-induced noise signals became approximately 9 times and 18 times that of the corrosion signals, respectively, significantly enhancing their impact on the corrosion signals. In the presence of both flow-induced noise and corrosion signals, the average amplitude of the mixed signals slightly increased due to the superposition of the two signal types.

**Figure 3:**
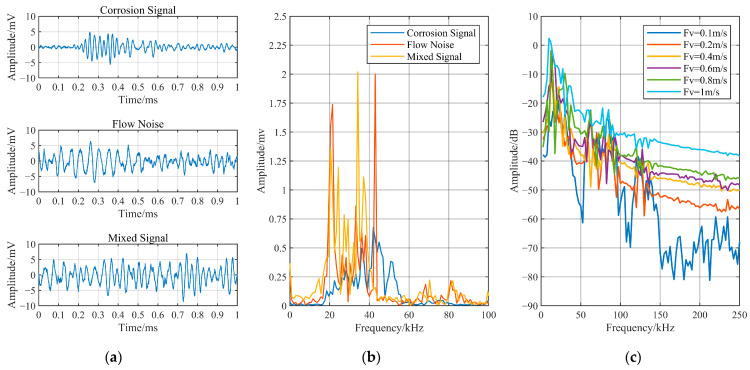
Time–frequency analysis of the original signals. (**a**) Waveforms of three types of signals; (**b**) spectrograms of three types of signals; (**c**) power spectra of flow-induced noise at different flow velocities.

Taking the data collected at a flow velocity of 0.1 m/s as an example, the waveforms of the three types of signals obtained during the experiment are shown in Figure 3a. The corrosion signal without noise interference appears as a burst-type AE signal, with a maximum amplitude close to that of flow-induced noise. The signal’s rise time is approximately 0.15 ms, its duration is approximately 0.4 ms, and the maximum amplitude is 5.6 mV. Flow-induced noise and mixed signals exhibit sustained-type AE signals, making it challenging to discern independent corrosion pulse components from mixed signals. The maximum amplitudes are slightly larger than that of the corrosion signal, measuring 7.2 mV and 9.1 mV, respectively. The spectra of the three signal types are illustrated in Figure 3b. The frequency of the corrosion signal is mainly distributed between 20 kHz and 55 kHz, with a frequency distribution showing negative skewness. The flow-induced noise and the mixed signal are primarily composed of components with frequencies between 15 kHz and 45 kHz, along with secondary frequency bands between 60 kHz and 90 kHz. A spectral analysis of the flow-induced noise at different flow velocities revealed that as the flow velocity increases, the amplitude of the signal changes, but the frequency distribution of the flow-induced noise remains relatively consistent, as shown in Figure 3c. When conducting AE monitoring for pipeline corrosion during the flow of an internal medium, it is challenging to determine from the waveform and spectrum of the signal whether corrosion pulses are present. Therefore, effectively identifying corrosion signalling components from mixed signals is a prerequisite for conducting the AE monitoring of pipeline corrosion.

## 4. Results and Discussion

### 4.1. Blind Source Separation of Signals

**Figure 4:**
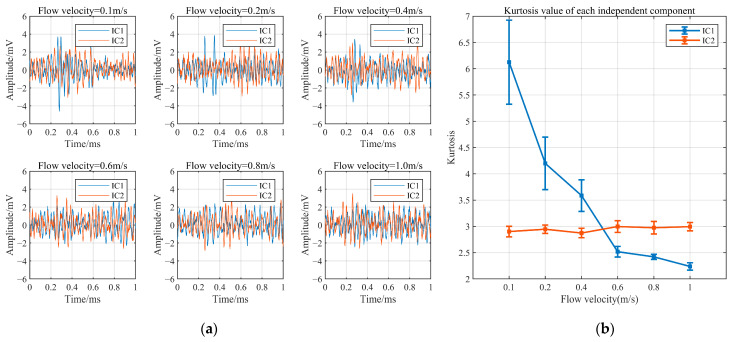
Blind source separation results for mixed signals at different flow velocities. (**a**) Separated waveforms; (**b**) kurtosis values for each IC.

BSS refers to the process of extracting and recovering individual source signals that cannot be observed from a set of mixed signals. In the AE monitoring of pipeline corrosion in the presence of flow-induced noise interference, the observed signal consists of a mixed signal of corrosion and flow-induced noise collected by sensors. SSA constructs a trajectory matrix based on the resonant behaviours associated with different specific frequencies during corrosion and flow processes. It decomposes and reconstructs the trajectory matrix to extract signals representing different components of the original time series. As the mixed signals in this study mainly involve two types of sources, corrosion signals and flow-induced noise, the singular value decomposition was set to 2 during the grouping process to separate the mixed signals into two observed signalling components. The singular spectrum analysis (SSA) is capable of separating different frequency components in a mixed signal. However, as corrosion signals and flow-induced signals contain identical frequency components, the independent component analysis (ICA) was employed for the secondary reconstruction of the two signalling components. By maximizing the non-Gaussianity of the signals, the independent time-domain representations of the corrosion signal and flow-induced noise signal were obtained. The reconstructed signals of both types were normalized to enhance the temporal characteristics of the corrosion signal. Signal separation using SSA-ICA was performed on mixed signals at different flow velocities, as shown in Figure 4. IC1 represents the reconstructed corrosion signal, and IC2 represents the reconstructed flow-induced noise. To evaluate the separation results of the mixed signals, the kurtosis index was employed, and its calculation formula is as follows:

(10) $$K=\frac{\frac{1}{n}\sum_{i=1}^{n}{\left(x_{i}-\bar{x}\right)}^{4}}{{\left(\frac{1}{n}\sum_{i=1}^{n}{\left(x_{i}-\bar{x}\right)}^{2}\right)}^{2}}$$

Kurtosis reflects the impulsive characteristics of a signal, with a kurtosis value of 3 indicating a normal distribution. Corrosion signals typically have distinct pulse components, and their kurtosis should be greater than 3. In contrast, flow-induced noise is a typical continuous signal, and its kurtosis should be approximately 3. The kurtosis values of IC1 and IC2 were calculated, as shown in Figure 4b. The kurtosis of the reconstructed flow-induced noise signals at different flow velocities is approximately 3, which is consistent with a normal distribution. At the flow velocities of 0.1 m/s, 0.2 m/s, and 0.4 m/s, where the average amplitude of the flow-induced noise is within three times that of the corrosion signal, the kurtosis values of the reconstructed corrosion signals are greater than 3, indicating a good separation effect. However, as the flow velocity increases to 0.6 m/s, 0.8 m/s, and 1.0 m/s, the kurtosis values of the reconstructed corrosion signals are less than 3, suggesting that at these flow velocities, flow-induced noise strongly interferes with the corrosion signal, making it challenging to effectively extract the corrosion signal from the mixed signals.

### 4.2. Feature Extraction and Model Training

**Figure 5:**
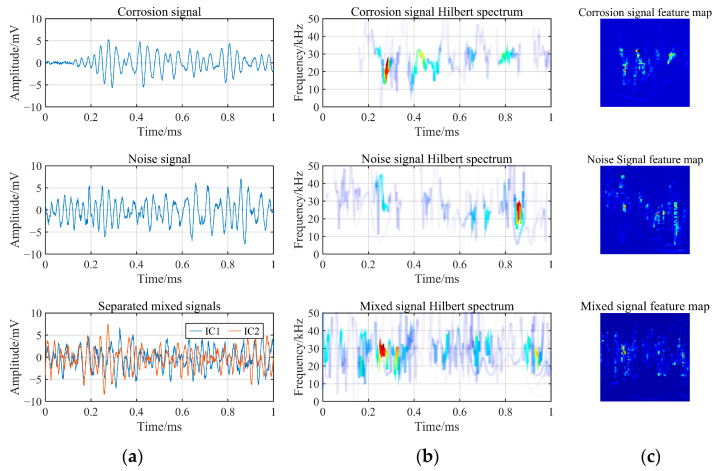
Construction of time–frequency feature maps for signals. (**a**) Original waveforms of corrosion signals, flow-induced noise, and separated waveforms of mixed signals; (**b**) Hilbert spectra of three types of signals; (**c**) compressed signal feature map.

After performing SSA-ICA on the mixed signals, the HHT was employed to obtain the Hilbert spectrum of each signal category. The Hilbert spectrum of the mixed signal reflects the coupling of IC1 and IC2, as shown in Figure 5a,b. The Hilbert spectrum is a three-dimensional spectrum involving time, frequency, and energy. To increase the computational efficiency of the subsequent CNN, the Hilbert spectrum was compressed to construct a feature map of size 56 × 56, which served as the input features for the CNN, as illustrated in Figure 5c.

The corrosion signals and flow-induced noise signals obtained during the experiment (labels 0–6) were subjected to HHT to construct feature maps. For the mixed signals obtained during the experiment (labels 7–12), signal separation was first performed, followed by HHT to construct feature maps. These feature maps were then divided into training and validation sets at a 3:1 ratio, consisting of 6000 training data points and 2000 validation data points for each label. The data were grouped based on different flow rates, resulting in six sets of training data, including corrosion signals, flow-induced noise at different flow rates, and mixed signals at different flow rates. These sets were input into the constructed CNN.

**Figure 6:**
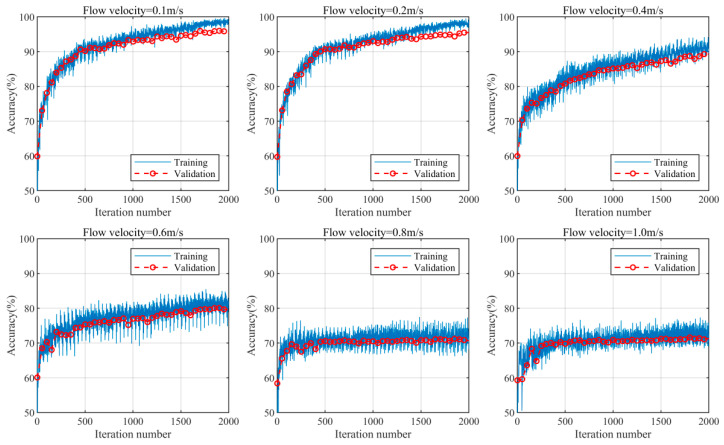
Accuracy graph of the SSA-ICA-CNN model iterations at different flow rates.

**Figure 7:**
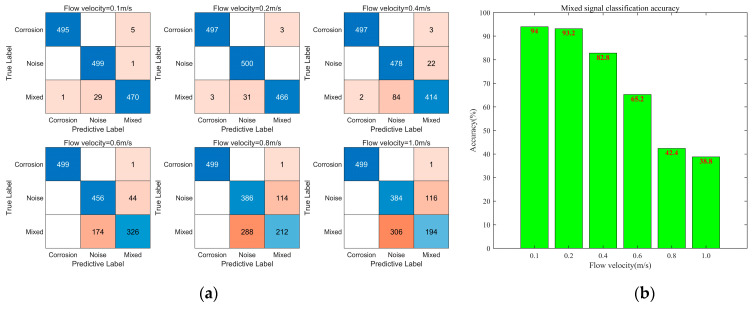
Recognition results at different flow rates. (**a**) Confusion matrices of model recognition results at various flow rates (The blue color is the correct recognition result, and darker blue indicates more correct recognition result. The orange color is the error recognition result, and darker orange indicates more error recognition result.); (**b**) recognition accuracy of mixed signals at different flow rates.

The recognition results of the AE signals under different flow rates are shown in Figure 6, with each dataset undergoing 2000 iterations of optimization. As the flow rate increases, the accuracy of the recognition model gradually decreases. The overall recognition accuracy decreases from nearly 98% (at a flow rate of 0.1 m/s) to 70% (at a flow rate of 1.0 m/s), which is consistent with the separation results of mixed signals in Section 4.1. This indicates that with an increasing flow velocity, the continuously enhanced flow-induced noise strongly interferes with the corrosion signal. At this point, the model struggles to distinguish whether the corrosion signalling component is present in the mixed signals. The results of a further analysis of the classification results of the test set at various flow rates are presented in Figure 7. Due to the distinct time–frequency characteristics of the corrosion signal itself compared to those of flow-induced noise and mixed signals, the corrosion signal can be accurately identified at different flow rates. However, as the flow velocity increases, the recognition accuracy of the flow-induced noise and mixed signals gradually decrease. In particular, when the flow velocity exceeds 0.6 m/s, the strong interference effect of the flow-induced noise on the corrosion signal causes the time–frequency characteristics of the mixed signals to approach those of the flow-induced noise. Consequently, most mixed signals are identified as flow-induced noise. At the flow velocities of 0.1 m/s, 0.2 m/s, and 0.4 m/s, the average amplitudes of the flow-induced noise are 1.5 times, 1.8 times, and 2.5 times that of the corrosion signal, respectively. The recognition accuracy of the mixed signals remains above 80%, reaching 94.0%, 93.2%, and 82.8%, respectively. However, at the flow velocities of 0.6 m/s, 0.8 m/s, and 1.0 m/s, the average amplitude of the flow-induced noise becomes 3.5 times, 8.3 times, and 17.8 times that of the corrosion signal, respectively, leading to a decrease in the recognition accuracy of the mixed signals to 65.2%, 42.4%, and 38.8%, respectively. In cases of excessive flow-induced noise, the effective identification of mixed signals becomes challenging.

### 4.3. Comparison of Model Effects

**Figure 8:**
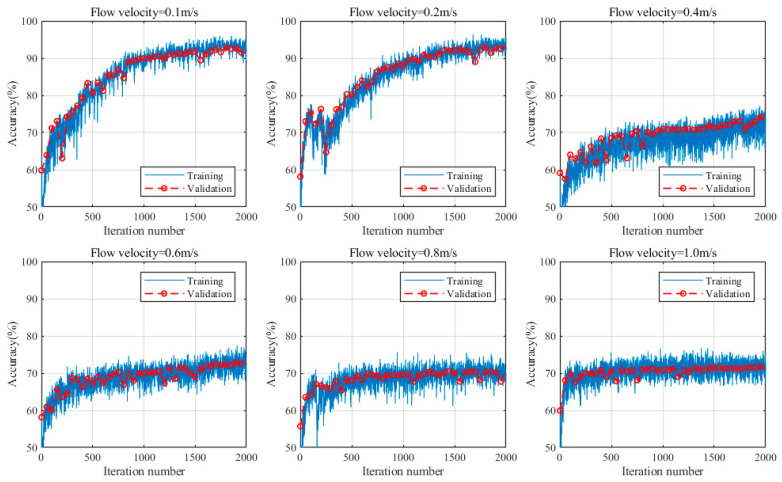
Accuracy of VMD-CNN model iterations at different flow rates.

The results of signal recognition indicate that the effectiveness of the separation method in the model directly influences the recognition accuracy of the subsequent CNN network. To highlight the advantages of the proposed model, variational mode decomposition (VMD) was used instead of SSA-ICA in the proposed model, and a comparison of the recognition performance was conducted. VMD is an adaptive, completely nonrecursive signal-processing method that decomposes time-series data into a series of IMFs with finite bandwidths [28]. VMD iteratively searches for the optimal solution of variational modes and is currently a popular method for nonstationary signal processing. The model’s iteration accuracy under different flow rates after using VMD for signal separation and employing the same feature-extraction method and CNN architecture is shown in Figure 8. When the flow rate is 0.1 m/s or 0.2 m/s, the VMD-CNN model can effectively distinguish three types of signals, with an average recognition accuracy of over 90%, which is slightly lower than that of the proposed SSA-ICA-CNN model. However, when the flow rate increases to 0.4 m/s and above, the recognition accuracy of the VMD-CNN is approximately 70%, making it difficult to effectively distinguish the three types of signals. This is because at lower flow rates, the frequency domain information of flow-induced noise and corrosion signals differs, and VMD can achieve the effective separation of mixed signals through precise frequency domain divisions. As the flow rate increases, the energy of the flow-induced noise in the same frequency band becomes stronger, obscuring the original frequency band of the corrosion signal. At this point, the separated signals obtained through VMD decomposition with different bandwidths essentially remain mixed signals of corrosion and flow-induced noise. Therefore, the subsequent CNN network struggles to distinguish between these signals. In contrast, the proposed SSA-ICA-CNN model comprehensively analyses the temporal and frequency domains of mixed signals, utilizes the frequency differences and non-Gaussian characteristics of signals for decomposition, and combines signal feature maps with deep learning networks, achieving better recognition results.

### 4.4. Hyperparameter Optimization Analysis

**Figure 9:**
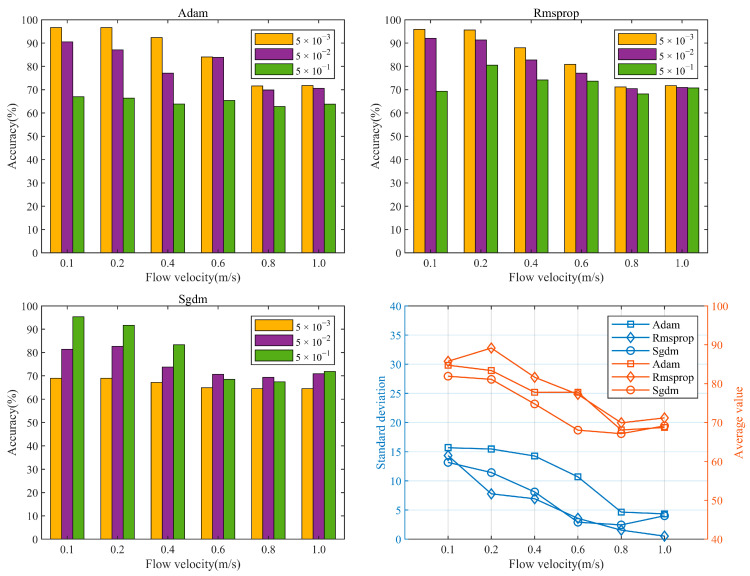
Performance comparison of different hyperparametric networks.

To investigate the impact of network hyperparameters on the accuracy of the proposed model, a comparative analysis was conducted on two categories of hyperparameters: deep learning optimizers and initial learning rates. The deep learning optimizers can adaptively adjust the parameters of the neural network model to maximize its potential. Currently, widely used optimizers include SGDM, RMSProp, and Adam [29]. The learning rate determines the magnitude of weight updates in each iteration, directly affecting the effectiveness of the training process. Learning rates that are too large or too small may result in training difficulties or slow convergence. Three types of optimizers and three different initial learning rates (0.005, 0.05, and 0.5) were compared to evaluate the network performance, as shown in Figure 9. When using the Adam and RMSProp optimizers, the highest recognition accuracy was achieved, with an initial learning rate of 0.005, which decreased as the learning rate increased. Conversely, when using the SGDM optimizer, the lowest recognition accuracy was observed, with an initial learning rate of 0.005. By computing the average recognition accuracy and standard deviations of each model under different flow rates, an evaluation of the network accuracy and robustness under different optimizers was performed. The network performance was found to be superior when using the RMSProp optimizer, exhibiting a high recognition accuracy and minimal fluctuations in response to changes in the learning rate. Therefore, it is recommended that the proposed model be configured with the RMSProp optimizer and an initial learning rate of 0.005.

## 5. Conclusions

This study explores the capability of applying machine learning to acoustic emission signals for identifying weak damage signals under strong noise backgrounds. The proposed model is the first to address the problem of separating and recognizing pipeline corrosion signals in the presence of flow-induced noise interference, providing a foundation for the subsequent online monitoring of pipeline corrosion. The findings of this study suggest that with appropriate signal analysis methods, the interference of noise can be mitigated to some extent, not only in corrosion acoustic emission monitoring but also in other fields where acoustic emission technology is commonly applied, such as fracture detection, fatigue detection, friction detection, and impact detection. This extends the application range of acoustic emission technology and effectively enhances its advantages in micro-damage monitoring. Based on simulated experiments in pipeline corrosion monitoring, the following conclusions can be drawn:

(1) Through a time–frequency domain analysis of corrosion signals, flow-induced noise signals, and mixed signals obtained in the experiments, it was found that flow-induced noise interfered with acoustic sources of pipeline corrosion. The similarity in the time and frequency domains between signals caused by pipeline corrosion and flow-induced noise makes it challenging to identify acoustic sources of corrosion from traditional AE parameters or waveforms.
(2) By comparing the separation effects of mixed signals at different flow rates, it was observed that when the average amplitude of the flow-induced noise was within three times that of the corrosion signal, the separation of two independent acoustic sources in mixed signals was more effective. Based on the accuracy results of the subsequent recognition model, the separation module plays a critical role in identifying corrosion signals under the interference of flow-induced noise. However, the intensity range of the noise must still be considered, as an excessively strong noise can continue to interfere with the corrosion signals.
(3) The impacts of different separation algorithms, SSA-ICA and VMD, as well as two types of hyperparameters, deep learning optimizers and initial learning rates, on network performance were individually compared. The results indicate that separation algorithms are more critical for recognition effectiveness, while hyperparameter optimization can also improve model performance to some extent. Additionally, the model demonstrated excellent recognition capabilities for corrosion signals without noise interference at different flow rates, making it equally suitable for pipeline corrosion monitoring during shutdown periods.

In future work, further optimization of the algorithm will be pursued to enhance the separation and identification capabilities and to explore the capability of separating and recognizing corrosion signals under different noise backgrounds. Moreover, on the basis of accurate corrosion signal identification, future studies should include the rapid dimensionality reduction in massive monitoring data, the optimization of multi-model fusion recognition algorithms, and the development of corrosion evolution prediction models to achieve comprehensive monitoring and assessments of pipeline health throughout a lifecycle.

## Figures and Tables

**Table 1:** CNN model parameters.

Name	Filters	Kernal Size/Stride	Input Size	Output Size	Parameter Scale
Conv_1	8	8/1	1@56 × 56	8@56 × 56	520
Pooling_1		2/2	8@56 × 56	16@28 × 28	-
Conv_2	16	8/1	16@28 × 28	16@14 × 14	8208
Pooling_2		2/2	16@14 × 14	32@14 × 14	-
Conv_3	32	6/1	32@14 × 14	32@7 × 7	18,464
Pooling_3		2/2	32@7 × 7	64@7 × 7	—
Conv_4	64	3/1	64@7 × 7	64@3 × 3	18,496
Pooling_4		2/2	64@3 × 3	3@1 × 1	—

**Table 2:** Generated dataset details.

Pipeline Status	Flow Rate (m/s)	Label	Signal Length	Average Signal Amplitude (V)
Corrosion	0	0	1024	0.0013
Corrosion free	0.1	1	1024	0.0020
Corrosion free	0.2	2	1024	0.0024
Corrosion free	0.4	3	1024	0.0032
Corrosion free	0.6	4	1024	0.0045
Corrosion free	0.8	5	1024	0.0108
Corrosion free	1.0	6	1024	0.0231
Corrosion	0.1	7	1024	0.0024
Corrosion	0.2	8	1024	0.0028
Corrosion	0.4	9	1024	0.0033
Corrosion	0.6	10	1024	0.0046
Corrosion	0.8	11	1024	0.0109
Corrosion	1.0	12	1024	0.0232

## Data Availability

The raw data supporting the conclusions of this article will be made available by the authors on request.
